# Gender Differences in the Association between Dietary Pattern and the Incidence of Hypertension in Middle-Aged and Older Adults

**DOI:** 10.3390/nu10020252

**Published:** 2018-02-23

**Authors:** SuJin Song, Jiwon Kim, Jihye Kim

**Affiliations:** 1 Department of Food and Nutrition, Hannam University, Daejeon 34054, Korea; sjsong@hnu.kr; 2 Department of Medical Nutrition, Graduate School of East-West Medical Science, Kyung Hee University, Yongin 17104, Korea; jiwonns@khu.ac.kr

**Keywords:** whole grain and legumes pattern, hypertension, gender difference, Korean adult

## Abstract

We examined gender differences in the association between dietary patterns and the risk of hypertension, using the Korean Genome and Epidemiology Study data. A total of 5090 participants (2457 men and 2633 women) aged 40–69 years without hypertension at baseline were selected. Dietary patterns were obtained using factor analysis based on 26 food groups, evaluated by a validated semi-quantitative food frequency questionnaire at baseline. Hypertension was defined as systolic blood pressure ≥ 140 mmHg or diastolic blood pressure ≥ 90 mmHg, or the use of antihypertensive medication using the biennial measurements. Multivariate Cox proportional hazards models were used to examine the associations between dietary patterns and hypertension. Four dietary patterns were extracted: coffee, fat, and sweets; prudent; whole grains and legumes; and traditional (men)/Western (women). Women in the highest tertile of the whole grains and legumes pattern scores showed a lower risk of incident hypertension compared with those in the lowest tertile (hazard ratio = 0.77, 95% confidence interval = 0.59–1.00, *p*-trend = 0.048). Other dietary patterns were not associated with hypertension in either men or women. A diet rich in whole grains and legumes is inversely associated with the risk of hypertension in Korean women, suggesting a gender difference in the association between diet and hypertension.

## 1. Introduction

Hypertension is a major public health issue worldwide [1], and it is closely associated with increased risks of morbidity and mortality in cardiovascular and kidney diseases [1,2]. According to the Korea National Health and Nutrition Examination Survey data, the prevalence of hypertension in Korean adults has increased from 26.5% in men and 23.8% in women in 2007, to 35.1% in men and 29.1% in women in 2015 [3].

Diet is an important determinant of hypertension development. A prudent dietary pattern has shown an inverse association with elevated blood pressure. In particular, the Dietary Approaches to Stop Hypertension (DASH) eating pattern, which emphasizes higher consumption of whole grains, legumes, nuts, fruits, vegetables, and low-fat dairy products, and lower consumption of fat, sweets, and refined grains, has been shown to have a favorable effect on blood pressure in epidemiologic studies [4,5,6,7]. In contrast, a Western-style dietary pattern, which consists of refined grains, red/processed meat, and high fat foods, has been associated with an increased risk of hypertension [8,9,10].

A few studies regarding dietary patterns associated with hypertension have been conducted in Asian populations [11,12,13]. In a cross-sectional study, a typical traditional diet, characterized by higher intakes of pork, egg, soybean, vegetables, fruits, and nuts, was inversely associated with hypertension in Chinese adults [13]. Among Japanese women aged 40–69 years, a diet pattern with higher consumption of vegetables, potatoes, fruits, beans, and seaweeds was related to lower blood pressure levels [11]. Recent studies have also suggested gender differences in the relationships between dietary factors and risks of chronic diseases, such as metabolic syndrome and hypertension [14].

As dietary patterns and their relationships with hypertension vary according to gender, age, and ethnicity, investigating population-specific dietary patterns associated with hypertension is important. In addition, gender differences in the association between dietary pattern and hypertension has not been investigated in a prospective study. Therefore, the aim of this study was to identify the associations of dietary patterns with the risk of incident hypertension in Korean adults and examine gender differences in the associations, using a large community-based cohort dataset.

## 2. Materials and Methods

### 2.1. Study Population

The Korean Genome and Epidemiology Study is a prospective, community-based cohort study, which has been conducted to explore lifestyle and genetic risk factors associated with chronic diseases, such as diabetes mellitus and hypertension, in Koreans. The cohort included 10,030 Korean adults, aged 40–69 years, who lived in the Ansan and Ansung areas. Baseline examinations were performed between 2001 and 2002 and follow-up examinations were performed every 2 years over a 6-year period (2001–2006). All participants completed questionnaires on demographic information, lifestyle, medical history, and health conditions at baseline; anthropometric and blood pressure measurements were conducted biennially.

Of these 10,030 participants, 3271 participants who had hypertension at baseline, 799 participants who refused to participate in the follow-up surveys, 110 participants who had cardiovascular disease or cancer, 202 participants who had implausible energy intakes (<500 kcal or >6000 kcal per day), and 425 participants who did not complete the food frequency questionnaire (FFQ) were excluded from the study. In addition, 133 participants were excluded because of missing data. Finally, a total of 5090 participants (2457 men and 2633 women) were included in the data analysis. The study protocol was approved by the Institutional Review Boards of the Korea Centers for Disease Control and Prevention and Kyung Hee University, and informed written consent was obtained from all participants.

### 2.2. Dietary Assessment

Dietary intake was assessed using a validated, 103-item, semi-quantitative FFQ [15] at baseline. In the FFQ, participants were asked how often and how much they consumed each food, on average, during the past year. Frequency was classified into nine categories: never/seldom, once/month, 2–3 times/month, 1–2 times/week, 3–4 times/week, 5–6 times/week, once/day, 2 times/day, or ≥3 times/day. The answer for portion size had three categories: small (1/2 serving), standard (1 serving), or large (≥2 servings).

To identify dietary patterns, food items were categorized into 26 food groups based on common food groups classified in the Korean nutrient database [16]. Since the grain and grain product intake of Koreans is very high, this group was divided into seven subgroups: white rice, whole grains, noodles, rice cakes, breads, pizza and hamburgers, and cereals and snacks. The vegetables group was divided into two subgroups: vegetables and kimchi (traditional fermented salted vegetables). Kimchi was presented as a single group because it is commonly eaten as a traditional side dish in Korea, but it has high sodium levels, unlike other vegetables. Coffee was also separated from the beverage group, because the consumption of coffee is very common between meals or after meals among Korean adults. Nutrient intake was calculated using the seventh edition of the Korean food composition table [17].

### 2.3. Definition of Hypertension

After resting in a seated position for at least 5 min, participants’ blood pressures were measured by trained technicians using a mercury sphygmomanometer (Baumanometer Standby; W.A. Baum Co. Inc., New York, NY, USA). Two readings of systolic blood pressure (SBP) and diastolic blood pressure (DBP) were recorded, and the average of the two readings was used for the analysis. Hypertension was defined as SBP ≥ 140 mmHg or DBP ≥ 90 mmHg or the use of antihypertensive medication, based on the 2003 guidelines of the Joint National Committee on Prevention, Detection, Evaluation, and Treatment of High Blood Pressure [2]. SBP and DBP were calculated at Korotkoff phase I and Korotkoff phase V, respectively.

### 2.4. Covariates

Demographic factors, socioeconomic status, and lifestyle factors of participants were determined using questionnaires by trained interviewers. Educational level was categorized into three groups: ≤6 years, 7–12 years, or >12 years. Monthly household income was categorized into four groups: less than 1 million Korean Won (approx. less than 850 US$ in 2016), 1 to less than 2 million Korean Won (approx. 850 to less than 1700 US$), 2 to less than 3 million Korean Won (approx. 1700 to less than 2500 US$), and greater than or equal to 3 million Korean Won. Smoking status was classified into three groups: never-smoker, former smoker, or current smoker. Alcohol consumption was classified into three groups: never-drinker, former drinker, or current drinker. Physical activity was assessed using metabolic equivalent of task (MET) hours per day. To obtain METs, participants reported hours spent on sleep and five types of activities, classified according to activity intensity, including sedentary, very light, light, moderate, and heavy activity. Total MET-hours per day were calculated by multiplying the reported hours spent on each type of activity during a day by the MET values that were determined based on the type or intensity of activity [18]. Height was measured to the nearest 0.1 cm using a stadiometer (Aluminum anthropometer; Samhwa Instrument, Seoul, Korea) and body weight was measured to 0.1 kg in light clothes, without shoes, using a bioelectrical impedance analysis (Inbody 3.0; Biospace Corp., Seoul, Korea) by trained research staff. Body mass index (BMI) was calculated as weight divided by height squared (kg/m^2^).

### 2.5. Statistical Analysis

Dietary patterns were derived for men and women separately using factor analysis. For the adjustment, the contribution of each food group to dietary pattern extraction was calculated as the energy intake consumed from each food divided by total energy intake consumed in a day. They were entered into the factor analysis using the FACTOR procedure of SAS software. To identify the number of factors to be retained, we used the eigenvalues > 1.3 criterion, the most widely used in factor analysis, as a first step. The factors were rotated by orthogonal transformation to achieve a simpler structure with greater interpretability. Post-rotated factor loadings revealed four factors that described the distinct dietary patterns of the study population. The scree plot showed a small break in the eigenvalues after four factors, which suggested that retention of four factors was appropriate for rotation. After varimax rotation, factor scores were saved from principle component analysis for each individual. The factor scores for each dietary pattern and for each individual were determined by summing the intake of each food group weighted by factor loading. The factor scores of each dietary pattern were categorized into tertiles and used for comparisons of nutrient intakes and other lifestyle variables.

Continuous variables were expressed as means and standard deviations, and categorical variables were expressed as percentages. Differences in baseline characteristics were examined by Chi-square tests or generalized linear model tests, as appropriate. We calculated the hazard ratios (HRs) and 95% confidence intervals (CIs) for incident hypertension across the tertiles of dietary pattern scores by time-dependent Cox proportional hazards models. In multivariable adjusted models, Model 1 was adjusted for age and BMI. Model 2 was adjusted for age, BMI, physical activity, educational level, household income, smoking status, and alcohol intake. All data analyses were conducted using SAS software, version 9.4 (SAS Institute, Cary, NC, USA). All *p*-values < 0.05 were considered statistically significant.

## 3. Results

### 3.1. Dietary Patterns in Men and Women

We identified dietary patterns by gender using factor analysis (Table 1). Different dietary patterns were derived between men and women. Among men, four dietary patterns were identified: a coffee, fat, and sweets pattern, with a higher intake of coffee with oils and fats, and sweets; a prudent pattern, with high factor loadings of vegetables, mushrooms, meat, fish, and seaweeds; a whole grains and legumes pattern; and a traditional pattern, with a high intake of Korean traditional foods, such as legumes, soybean paste, and kimchi. Women also showed four major dietary patterns: a coffee, fat, and sweets pattern, a prudent pattern, a whole grains and legumes pattern, and a Western pattern, characterized by a high intake of noodles, breads, pizza and hamburgers, cereals and snacks, and meat.

### 3.2. Baseline Characteristics by Dietary Pattern Score Tertile

Baseline characteristics across dietary pattern score tertiles by gender are presented in Table 2 and Table 3. In both men and women, individuals with a higher score on the coffee, fat, and sweets pattern were more likely to be younger, have a higher income, be more educated, smoke currently, and be physically inactive. The coffee, fat, and sweets pattern was positively associated with BMI and inversely associated with SBP in both men and women. Men and women in the highest tertile of the prudent pattern tended to be younger, have higher incomes and education levels, drink more, and be less physically active than those in the lowest tertile. The prudent pattern was positively associated with BMI in men and inversely associated with blood pressure in women. Among men, the whole grains and legumes pattern was significantly associated with age, smoking status, and alcohol intake. Women who had a higher score for the whole grain and legume pattern were more likely to be older, have higher incomes and education levels, drink less, and have a lower DBP. The traditional pattern identified in men was positively associated with age, current smoking, physical activity, and blood pressure, but inversely associated with income, education, and BMI. The Western pattern observed in women was positively associated with income, education, and current alcohol drinking, but inversely associated with age, physical activity, and blood pressure.

### 3.3. Nutrient Intake by Dietary Pattern Score Tertile

As shown in Table 4 and Table 5, in both men and women, the coffee, fat, and sweets pattern was positively associated with a higher intake of fat, whereas the prudent pattern was associated with a lower intake of carbohydrates. Individuals in the highest tertile of the whole grains and legumes pattern had higher intakes of fiber, potassium and calcium, but a lower intake of carbohydrates. Men who had a higher score on the traditional pattern had higher intakes of carbohydrates, fiber, sodium, vitamin A, carotene, and vitamin C, but lower intakes of fat, protein, and calcium. Women in the highest tertile of the Western pattern had higher intakes of energy, fat, protein, potassium, and calcium, but lower intakes of carbohydrates, fiber, sodium, carotene, and vitamin C.

### 3.4. Association between Dietary Patterns and the Risk of Incident Hypertension

A follow-up rate of 75.3% was achieved. The average follow-up period was 41 months. During the 6 years of follow-up, we ascertained 361 and 342 cases of hypertension in men and women, respectively. Table 6 presents HRs and 95% CIs for the risk of incident hypertension across tertiles of dietary pattern scores by gender. Dietary patterns were not associated with the risk of hypertension in men. Women in the highest tertile of the whole grains and legumes pattern showed a lower risk of incident hypertension, compared with those in the lowest tertile of the whole grains and legumes pattern, after adjustment for age and BMI (HR for T3 vs. T1 = 0.75, 95% CI = 0.58–0.97, *p* for trend = 0.029). This trend remained after adjustment for age, BMI, income, education, alcohol intake, smoking, and physical activity (HR for T3 vs. T1 = 0.77, 95% CI = 0.59–1.00, *p* for trend = 0.048). Other dietary patterns did not show significant associations with the risk of hypertension in women.

## 4. Discussion

In a large scale, prospective study, we found different dietary patterns according to gender among middle-aged and older Korean adults. The dietary patterns for men were coffee, fat, and sweets, prudent, whole grains and legumes, and traditional patterns, whereas women had a Western pattern instead of the traditional pattern. Among these dietary patterns, the whole grains and legumes pattern was associated with a 23% lower risk of incident hypertension in women, after adjustment for potential confounders, such as age, BMI, income, education, alcohol intake, smoking, and physical activity. This significant association was not observed in men. Other dietary patterns were not associated with hypertension in either men or women.

Consistent with our findings, previous studies support an inverse relationship between dietary patterns rich in whole grains and legumes and hypertension. In the Women’s Health Study, women who consumed ≥4 servings/day of whole grains had a 23% lower risk of hypertension relative to those who consumed <0.5 servings/day of whole grains, after adjustment for lifestyle, clinical, and dietary factors, over a follow-up period of 10 years [19]. A randomized clinical trial reported that women consuming a whole grain and legume diet containing barley products, brown beans, and chickpeas for 4 weeks showed a significant reduction in blood pressure relative to a control group (−3 mmHg vs. 0 mmHg) in Swedish women aged 50–72 years [20]. A cross-sectional study of Iranian adults aged ≥19 years showed that the prevalence of elevated blood pressure was significantly lower among frequent legume consumers (≥3 times/week) compared with those who did not consume legumes (31.3% vs. 43.5%) [21].

The favorable effect of the whole grains and legumes pattern on hypertension might be due to combined and synergic effects between various nutrients and food components [22,23]. Nutrients included in whole grains and legumes, such as dietary fiber, calcium, potassium, and magnesium, have been shown to be helpful for reducing blood pressure [24,25,26,27]. A randomized, controlled trial showed that a diet rich in high-fiber plant foods, including dietary fiber (54 g/day) and beta-glucan (4.9 g/day), for 6 weeks significantly reduced SBP by 6.6 mmHg compared with a control diet, among Swedish adults aged 25–65 years [28]. Potassium or magnesium in diets directly affects blood pressure levels by controlling the contraction or vasodilation of vascular cells [25,29].

The components of whole grains and legumes might have a beneficial effect on hypertension by mediating vascular endothelial functions [30]. Jenkins and colleagues reported that a whole grain diet was associated with a lower reactive hyperemia index, a surrogate of peripheral vascular function, relative to a control diet [31]. In the Coronary Artery Risk Development of Young Adults study, a diet characterized by a higher intake of whole grains and legumes was inversely related to specific cellular adhesion molecules, which are known as biomarkers of endothelial dysfunction [32]. In addition to this, whole grains and legumes may moderate blood pressure through improving insulin sensitivity [33] and/or inducing an increase in anti-inflammatory markers, such as adiponectin [22]. Recently, gut microbiota and its metabolites have been known to influence the regulation of blood pressure. Short chain fatty acids (SCFA), derived from the gut microbial fermentation of dietary fiber, may regulate blood pressure via SCFA receptors. For example, the stimulation of G protein-coupled receptor 41, a SCFA receptor, decreases blood pressure by inducing vasorelaxation [34,35].

Interestingly, the inverse association of the whole grains and legumes pattern with hypertension was found only in women. The gender difference in the association between dietary pattern and hypertension has not been clearly explained. Diet might influence hypertension between men and women differently due to the renin-angiotensin system, sex hormones, or sex-specific genes, and increased inflammatory markers, which are related to the control of blood pressure. The response to stimulation of the renin-angiotensin system may differ between men and women [36]. This may be related to sex hormones. For example, the decline in estrogen activates the renin-angiotensin system and increases angiotensin II levels; these alterations are related to elevated blood pressure [37]. Likewise, the effects of diet or nutrients on blood pressure may be greater in women than men. In a study of the Korean National Health and Nutrition Examination Survey, women who consumed fried foods frequently (≥2 times/week) had a 2.4-fold higher risk of hypertension compared with those who rarely consumed fried foods, whereas this significant association was not shown in men [38]. The reduction in blood pressure with a diet of reduced sodium, potassium supplementation or dark chocolate intervention was greater in women than men [39,40,41]. A modified lifestyle approach involving a healthy diet was more effective in alleviating risk factors of hypertension in women than in men [37]. Furthermore, there are many different pathophysiological and social reasons why the results may have been only significant in women, which could not be specifically determined in the present study. Therefore, the present study suggests that gender-based differences should be considered in the prevention and management of hypertension. Further studies are needed to elucidate underlying mechanisms, to explain gender differences in the association between diet and hypertension.

This study has several limitations. The study participants may not be representative of the entire Korean population, as this study was only conducted among middle-aged and older Korean adults. In addition, the procedures of factor analysis require several arbitrary decisions to identify dietary patterns. Despite these limitations, this is the first prospective study to evaluate the role of dietary patterns in the development of hypertension among Korean adults using large-scale cohort data. These findings suggest that a specific dietary pattern may be an independent risk factor for developing hypertension in Korean adults, and also indicates a gender difference in the association between dietary patterns and hypertension risk.

## 5. Conclusions

A diet rich in whole grains and legumes was inversely associated with the risk of developing hypertension in Korean women, suggesting a gender difference in the association between diet and hypertension. The findings from this study might be useful in suggesting dietary recommendations for the prevention and management of hypertension in this population. Future studies are needed to explore the underlying mechanism, to explain the association between dietary patterns and hypertension.

## Figures and Tables

**Table 1:** Factor loading matrix for major dietary patterns of Korean adults by gender ^1^.

Food or Food Group	Coffee, Fat, and Sweets Pattern (M/W)	Prudent Pattern (M/W)	Whole Grains and Legumes Pattern (M/W)	Traditional (M)/Western Pattern (W)
White rice			−0.84/−0.89	
Whole grains			0.90/0.93	
Noodles				−0.25/0.32
Rice cakes				
Breads				−0.47/0.57
Pizza and hamburgers				−0.46/0.45
Cereals and snacks				−0.37/0.44
Oils and fats	0.90/0.92			−0.32/0.08
Sweets	0.95/0.95			
Potato and sweet potato		0.31/−0.06		
Legumes			0.45/0.43	0.33/−0.25
Starch jelly		0.30/0.36		
Soybean paste				0.54/−0.47
Nuts				
Kimchi				0.50/−0.50
Vegetables		0.55/0.56		
Mushrooms		0.55/0.52		
Fruits				
Meat		0.33/0.33		−0.09/0.35
Eggs				
Fish		0.65/0.57		
Seaweeds		0.45/0.39		
Milk and dairy products				
Coffee	0.91/0.92			−0.35/0.02
Carbonated drinks				
Other beverages		0.41/0.35		−0.33/0.04

M, men; W, women. ^1^ Only food groups with absolute value of factor loading ≥ |0.30| for each dietary pattern are presented for simplicity.

**Table 2:** Characteristics of the study participants according to the tertiles (T) of dietary pattern scores in men ^1,2^.

Men	Coffee, Fat, and Sweets Pattern			Prudent Pattern			Whole Grains and Legumes Pattern			Traditional Pattern		
	T1	T2	T3	T1	T2	T3	T1	T2	T3	T1	T2	T3
*n* (2457)	819	819	819	819	819	819	819	819	819	819	819	819
Age (years)	51.8 (8.8)	49.2 (7.9)	49.8 *** (8.2)	52.1 (8.9)	50.0 (8.2)	48.8 (7.7)	49.4 (8.1)	50.4 (8.4)	51.1 ** (8.5)	48.2 (7.4)	49.6 (8.0)	53.1 *** (8.9)
Income (Korean Won/month) (%)												
<1 million	31.5	17.5	20.7 ***	35.5	19.4	14.7 ***	24.6	25.1	20.0	13.7	21.5	34.4 ***
1–2 million	29.4	31.7	30.4	33.7	29.0	28.8	30.5	30.8	30.1	30.6	29.2	31.6
2–3 million	17.8	24.0	22.3	15.9	25.1	23.0	21.9	20.4	21.7	23.4	23.2	17.4
≥3 million	21.4	26.9	26.7	14.9	26.5	33.5	23.0	23.7	28.3	32.3	26.0	16.7
Education (%)												
≤6 years	21.9	12.2	16.3 ***	25.3	14.1	11.0 ***	16.4	18.7	15.3	8.6	14.7	27.2 ***
7–12 years	57.3	61.0	60.5	57.4	61.9	59.5	61.8	56.5	60.5	59.6	61.9	57.3
>12 years	20.8	26.8	23.3	17.3	24.1	29.5	21.8	24.8	24.2	31.8	23.5	15.5
Smoking (%)												
Never	27.2	17.9	10.9 ***	18.0	18.5	19.5	17.8	17.0	21.2 **	20.1	19.3	16.5 *
Former	33.1	30.8	27.8	27.2	32.8	31.7	27.5	29.8	34.3	33.7	29.8	28.2
Current	39.8	51.3	61.3	54.7	48.8	48.8	54.7	53.2	44.6	30.3	50.9	55.3
Alcohol (%)												
Never	21.9	15.9	22.2 *	20.9	20.8	18.2 *	18.4	22.9	18.7 *	20.6	19.4	20.0
Former	9.1	8.2	10.7	12.0	7.7	8.2	7.7	9.5	10.7	9.9	9.5	8.5
Current	69.0	75.9	67.2	67.1	71.5	73.6	73.9	67.6	70.6	69.5	71.0	71.6
Physical activity (MET h/day)	25.8 (16.1)	23.0 (14.4)	21.3 *** (13.8)	26.1 (16.0)	22.3 (14.1)	21.7 *** (14.1)	23.7 (15.0)	23.3 (15.4)	23.2 (14.3)	21.5 (13.6)	22.6 (14.4)	26.1 *** (16.2)
BMI (kg/m^2^)	23.6 (2.7)	24.3 (2.9)	23.9 *** (2.9)	23.5 (2.9)	24.0 (2.8)	24.3 *** (2.8)	23.8 (2.8)	24.0 (3.0)	24.0 (2.7)	24.2 (2.8)	23.9 (2.9)	23.6 *** (2.8)
SBP (mmHg)	115.0 (11)	113.0 (11)	113.0 * (11)	114.0 (11)	113.0 (11)	113.0 (11)	114.0 (11)	113.0 (11)	114.0 (11)	113.0 (11)	114.0 (11)	114.0 * (11)
DBP (mmHg)	76.7 (7.3)	76.6 (7.5)	76.3 (7.4)	76.5 (6.8)	76.5 (7.7)	76.6 (7.7)	76.3 (7.3)	76.6 (7.5)	76.7 (7.5)	76.0 (7.6)	76.9 (7.6)	76.7 * (7.1)

BMI, body mass index; DBP, diastolic blood pressure; MET, Metabolic equivalent of task; SBP, systolic blood pressure. ^1^ Continuous variables are expressed as means (standard deviations) and categorical variables are expressed as percentages. ^2^ Generalized linear model tests for continuous variables and Chi-square tests for categorical variables were used to test differences in variables across the tertiles of dietary pattern scores. * *p* < 0.05, ** *p* < 0.001, *** *p* < 0.0001.

**Table 3:** Characteristics of the study participants according to the tertiles (T) of dietary pattern scores in women ^1,2^.

Women	Coffee, Fat, and Sweets Pattern			Prudent Pattern			Whole Grains and Legumes Pattern			Western Pattern		
	T1	T2	T3	T1	T2	T3	T1	T2	T3	T1	T2	T3
*n* (2633)	878	877	878	878	877	878	878	878	877	878	878	877
Age (years)	52.1 (8.7)	49.7 (8.3)	49.0 *** (8.0)	52.0 (9.0)	49.6 (8.1)	49.1 *** (7.7)	50.2 (8.5)	49.3 (8.2)	51.2 *** (8.4)	53.7 (8.7)	49.9 (8.2)	47.1 *** (6.8)
Income (Korean Won/month) (%)												
<1 million	44.2	26.1	28.5 ***	45.0	28.3	25.6 ***	38.8	29.0	31.1 **	49.5	29.9	19.4 ***
1–2 million	29.1	29.6	30.4	28.5	30.3	30.3	29.2	29.3	30.5	28.8	30.4	29.9
2–3 million	16.0	23.0	22.0	14.6	24.1	22.3	17.8	21.7	21.5	12.8	23.2	25.0
≥3 million	10.7	21.4	19.1	11.9	17.4	21.9	14.2	20.0	17.0	8.9	16.6	25.7
Education (%)												
≤6 years	44.5	32.3	30.0 ***	49.7	29.9	27.3 ***	41.1	30.0	35.7 ***	56.1	31.4	19.4 ***
7–12 years	50.7	58.9	60.2	46.0	61.0	62.8	53.7	58.6	57.5	40.8	61.6	67.4
>12 years	4.8	8.8	9.8	4.3	9.1	9.9	5.3	11.4	6.7	3.2	7.0	13.2
Smoking (%)												
Never	96.7	96.5	92.7 **	94.6	96.0	95.2	94.8	95.4	95.7	94.6	96.0	95.2
Former	0.7	0.9	1.9	1.9	0.7	0.9	1.5	1.0	1.0	1.0	1.1	1.5
Current	2.6	2.6	5.4	3.4	3.3	3.9	3.8	3.5	3.3	4.3	2.9	3.4
Alcohol (%)												
Never	73.6	66.8	64.2 ***	71.1	69.5	63.9 *	67.8	63.9	72.8 *	75.0	68.7	60.8 ***
Former	3.5	2.9	1.1	2.4	2.1	3.1	2.4	3.2	1.9	2.9	3.1	1.6
Current	22.8	30.4	34.7	26.5	28.4	33.0	29.8	32.9	25.2	22.2	28.2	37.6
Physical activity (MET h/day)	23.8 (15.4)	22.3 (13.7)	21.1 * (13.3)	24.0 (15.1)	21.6 (13.7)	21.7 ** (13.4)	22.9 (14.8)	22.3 (14.5)	22.1 (13.1)	25.1 (15.6)	22.1 (14.0)	20.1 *** (12.3)
BMI (kg/m^2^)	24.1 (3.1)	24.5 (3.1)	24.4 * (3.0)	24.2 (3.2)	24.4 (3.0)	24.4 (3.0)	24.3 (3.0)	24.3 (3.1)	24.4 (3.1)	24.4 (3.2)	24.5 (3.0)	24.1 (3.1)
SBP (mmHg)	112.0 (12)	111.0 (12)	110.0 ** (12)	112.0 (12)	111.0 (12)	110.0 * (12)	112.0 (11)	110.0 (12)	111.0 (12)	113.0 (12)	111.0 (12)	109.0 *** (12)
DBP (mmHg)	73.8 (8.1)	73.4 (8.0)	72.8 * (8.3)	74.0 (7.9)	73.0 (8.2)	73.0 * (8.4)	73.9 (7.8)	72.8 (8.4)	73.3 * (8.2)	74.4 (8.0)	73.3 (8.0)	72.2 *** (8.4)

BMI, body mass index; DBP, diastolic blood pressure; MET, Metabolic equivalent of task; SBP, systolic blood pressure. ^1^ Continuous variables are expressed as means (standard deviations) and categorical variables are expressed as percentages. ^2^ Generalized linear model tests for continuous variables and Chi-square tests for categorical variables were used to test differences in variables across the tertiles of dietary pattern scores. * *p* < 0.05, ** *p* < 0.001, *** *p* < 0.0001.

**Table 4:** Daily nutrient intake of the study participants across the tertiles (T) of dietary pattern scores in men ^1,2^.

Men	Coffee, Fat, and Sweet Patterns			Prudent Pattern			Whole Grains and Legumes Pattern			Traditional Pattern		
	T1	T2	T3	T1	T2	T3	T1	T2	T3	T1	T2	T3
*n* (2457)	819	819	819	819	819	819	819	819	819	819	819	819
Energy (kcal/day)	2086 (720)	2139 (605)	1984 *** (511)	1907 (618)	2078 (553)	2225 *** (649)	1973 (587)	2201 (685)	2036 *** (564)	2232 (663)	2085 (616)	1892 *** (532)
Carbohydrate (% of energy)	71.2 (7.3)	69.3 (6.8)	70.4 *** (6.5)	75.3 (5.2)	69.7 (5.4)	65.9 *** (6.5)	71.5 (7.7)	69.4 (7.1)	70.0 *** (5.6)	67.9 (6.3)	70.5 (6.7)	72.6 *** (6.9)
Protein (% of energy)	13.8 (2.4)	14.1 (2.3)	13.5 *** (2.1)	12.0 (1.4)	13.8 (1.5)	15.5 *** (2.2)	13.3 (2.4)	13.9 (2.4)	14.2 *** (1.9)	14.0 (2.1)	13.8 (2.3)	13.7 * (2.4)
Fat (% of energy)	14.9 (5.3)	16.6 (5.0)	16.1 *** (4.8)	12.6 (4.2)	16.4 (4.4)	18.6 *** (4.8)	15.2 (5.7)	16.7 (5.2)	15.8 *** (4.2)	18.2 (4.7)	15.8 (4.7)	13.7 *** (4.8)
Fiber (g/day)	7.4 (3.8)	6.8 (2.9)	6.2 *** (2.5)	5.7 (2.9)	6.8 (2.8)	7.9 *** (3.3)	5.5 (2.5)	7.1 (3.3)	7.8 *** (3.0)	6.4 (2.6)	6.7 (3.1)	7.3 *** (3.5)
Sodium (mg/day)	3466 (1936)	3420 (1558)	3172 ** (1377)	2742 (1490)	3388 (1520)	3927 *** (1699)	3155 (1614)	3608 (1759)	3296 *** (1521)	3135 (1502)	3154 (1418)	3770 *** (1896)
Potassium (mg/day)	2533 (1239)	2553 (984)	2507 (891)	1977 (868)	2527 (856)	3090 *** (1092)	2160 (903)	2733 (1140)	2700 *** (988)	2581 (1006)	2490 (1049)	2523 (1086)
Calcium (mg/day)	493 (276)	496 (241)	445 *** (215)	332 (175)	479 (195)	623 *** (266)	404 (223)	528 (264)	502 *** (232)	533 (265)	455 (229)	446 *** (233)
Vitamin A (µgRE/day)	576 (457)	576 (362)	499 *** (308)	361 (247)	541 (99)	749 *** (461)	491 (341)	611 (436)	548 *** (354)	548 (348)	525 (352)	578 * (438)
Carotene (µg/day)	2945 (2664)	2938 (2178)	2538 ** (1804)	1857 (1421)	2726 (811)	3838 *** (2816)	2509 (1959)	3112 (2592)	2800 *** (2112)	2648 (2014)	2669 (1982)	3104 *** (2662)
Vitamin C (mg/day)	132 (101)	118 (73)	108 *** (62)	91 (75)	120 (74)	147 *** (83)	103 (65)	137 (88)	118 *** (84)	110 (68)	120 (88)	128 *** (84)

^1^ All values are expressed as means (standard deviations). ^2^ Generalized linear model tests were used to test differences in daily nutrient intakes across the tertiles of dietary pattern scores. * *p* < 0.05, ** *p* < 0.001, *** *p* < 0.0001.

**Table 5:** Daily nutrient intake of the study participants across the tertiles (T) of dietary pattern scores in women ^1,2^.

Women	Coffee, Fat, and Sweets Pattern			Prudent Pattern			Whole Grains and Legumes Pattern			Western Pattern		
	T1	T2	T3	T1	T2	T3	T1	T2	T3	T1	T2	T3
*n* (2633)	878	877	878	878	877	878	877	878	878	878	877	878
Energy (kcal/day)	1958 (761)	1988 (613)	1790 *** (552)	1756 (567)	1939 (574)	2052 *** (767)	1826 (568)	2158 (825)	1763 *** (436)	1729 (616)	1903 (600)	2115 *** (686)
Carbohydrate (% of energy)	74.0 (7.5)	70.8 (6.9)	71.8 *** (6.6)	77.1 (5.2)	71.9 (5.5)	67.5 *** (7.0)	74.0 (7.7)	69.6 (7.6)	72.9 *** (5.0)	76.1 (6.1)	72.8 (5.9)	67.7 *** (6.7)
Protein (% of energy)	13.1 (2.4)	14.1 (2.3)	13.5 *** (2.2)	11.9 (1.4)	13.6 (1.5)	15.4 *** (2.4)	12.8 (2.4)	14.3 (2.4)	13.8 *** (1.8)	13.1 (2.5)	13.5 (2.1)	14.2 *** (2.2)
Fat (% of energy)	12.8 (5.5)	15.1 (5.1)	14.7 *** (4.9)	11.0 (4.1)	14.5 (4.5)	17.1 *** (5.3)	13.2 (5.7)	16.2 (5.6)	13.3 *** (3.7)	10.8 (4.0)	13.7 (4.1)	18.1 *** (4.9)
Fiber (g/day)	7.3 (4.1)	7.5 (3.4)	6.4 *** (2.7)	5.7 (2.5)	7.1 (3.0)	8.4 *** (4.1)	5.8 (3.2)	8.1 (4.1)	7.3 *** (2.5)	7.6 (4.1)	6.9 (3.1)	6.7 *** (3.0)
Sodium (mg/day)	2909 (1619)	3243 (1546)	2912 ** (1371)	2458 (1303)	3014 (1326)	3591 *** (1693)	2863 (1470)	3374 (1650)	2826 *** (1376)	3342 (1740)	2808 (1365)	2913 *** (1383)
Potassium (mg/day)	2472 (1400)	2715 (1147)	2464 *** (985)	1901 (769)	2558 (957)	3193 *** (1395)	2187 (1153)	3023 (1376)	2442 *** (835)	2485 (1313)	2489 (1096)	2678 ** (1156)
Calcium (mg/day)	463 (295)	552 (256)	466 *** (244)	335 (169)	487 (221)	640 *** (302)	401 (224)	590 (307)	472 *** (229)	450 (284)	471 (233)	541 *** (275)
Vitamin A (µgRE/day)	515 (466)	566 (384)	485 ** (314)	322 (221)	506 (307)	738 *** (485)	464 (338)	649 (480)	452 *** (312)	534 (468)	493 (342)	538 * (359)
Carotene (µg/day)	2650 (2781)	2844 (2200)	2434 ** (1782)	1628 (1270)	2527 (1865)	3773 *** (2901)	2345 (1844)	3288 (2886)	2297 *** (1866)	2855 (2727)	2510 (1999)	2564 * (2082)
Vitamin C (mg/day)	143 (131)	140 (96)	118 *** (77)	88 (59)	136 (93)	177 *** (129)	129 (111)	167 (123)	105 *** (56)	141 (122)	131 (97)	128 * (91)

^1^ All values are expressed as means (standard deviations). ^2^ Generalized linear model tests were used to test differences in daily nutrient intakes across the tertiles of dietary pattern scores. *p < 0.05, ** *p* < 0.001, *** *p* < 0.0001.

**Table 6:** Hazard ratios (HRs) and 95% confidence intervals (CIs) for the risk of incident hypertension across the tertiles (T) of dietary pattern scores by gender ^1^.

**Gender**	**Coffee, Fat, and Sweets Pattern**			***p* for Trend**	**Prudent Pattern**			***p* for Trend**
	**T1**	**T2**	**T3**		**T1**	**T2**	**T3**	
Men								
*n* (No. of cases)	819 (115)	819 (132)	819 (114)		819 (123)	819 (107)	819 (131)	
Model 1 ^2^	1.00	1.15 (0.89–1.48)	1.01 (0.78–1.31)	0.9687	1.00	0.91 (0.70–1.19)	1.18 (0.92–1.51)	0.2166
Model 2 ^3^	1.00	1.16 (0.90–1.50)	1.01 (0.78–1.32)	0.9628	1.00	0.96 (0.74–1.25)	1.26 (0.97–1.62)	0.0839
Women								
*n* (No. of cases)	878 (141)	877 (98)	878 (103)		878 (139)	877 (101)	878 (102)	
Model 1	1.00	0.74 (0.58–0.96)	0.87 (0.67–1.12)	0.2253	1.00	0.88 (0.68–1.14)	0.94 (0.72–1.21)	0.5945
Model 2	1.00	0.76 (0.59–0.99)	0.87 (0.67–1.13)	0.2491	1.00	0.91 (0.70–1.18)	0.98 (0.75–1.27)	0.8590
**Gender**	**Whole Grains and Legumes Pattern**			***p* for Trend**	**Traditional (Men)/Western (Women) Pattern**			***p* for Trend**
	**T1**	**T2**	**T3**		**T1**	**T2**	**T3**	
Men								
*n* (No. of cases)	819 (102)	819 (132)	819 (127)		819 (103)	819 (127)	819 (131)	
Model 1	1.00	1.19 (0.91–1.54)	1.14 (0.88–1.48)	0.3719	1.00	1.25 (0.97–1.63)	1.19 (0.91–1.55)	0.2313
Model 2	1.00	1.21 (0.93–1.57)	1.18 (0.91–1.54)	0.2376	1.00	1.22 (0.94–1.58)	1.10 (0.84–1.44)	0.5374
Women								
*n* (No. of cases)	878 (124)	877 (111)	878 (107)		878 (141)	877 (112)	878 (89)	
Model 1	1.00	0.93 (0.72–1.20)	0.75 (0.58–0.97)	0.0292	1.00	1.05 (0.82–1.35)	1.07 (0.80–1.42)	0.6091
Model 2	1.00	0.96 (0.74–1.24)	0.77 (0.59–1.00)	0.0480	1.00	1.10 (0.85–1.42)	1.15 (0.86–1.54)	0.3047

^1^ Time-dependent Cox proportional hazard models were used to calculate HRs (95% CIs) for the risk of incident hypertension across the tertiles of dietary pattern scores by gender. ^2^ Model 1 was adjusted for age and BMI. ^3^ Model 2 was adjusted for age, BMI, physical activity, educational level, household income, smoking status, and alcohol intake.
